# Physician skin cancer screening among U.S. military veterans: Results from the National Health Interview Survey

**DOI:** 10.1371/journal.pone.0251785

**Published:** 2021-05-18

**Authors:** Elliot J. Coups, Baichen Xu, Carolyn J. Heckman, Sharon L. Manne, Jerod L. Stapleton

**Affiliations:** 1 Medical Data Analytics, Parsippany, NJ, United States of America; 2 Rutgers Cancer Institute of New Jersey, Rutgers, The State University of New Jersey, New Brunswick, NJ, United States of America; 3 Department of Medicine, Rutgers Robert Wood Johnson Medical School, Rutgers, The State University of New Jersey, New Brunswick, NJ, United States of America; 4 Department of Health Behavior, Society & Policy, Rutgers School of Public Health, Rutgers, The State University of New Jersey, Piscataway, NJ, United States of America; 5 Department of Health, Behavior & Society, University of Kentucky College of Public Health, Lexington, KY, United States of America; University of Zurich, SWITZERLAND

## Abstract

**Introduction:**

Although military veterans are at increased risk for skin cancer, little is known about the extent to which they have been screened for skin cancer. The study objective was to examine the prevalence and correlates of physician skin cancer screening among U.S. military veterans.

**Methods:**

Data were drawn from the National Health Interview Survey. The study sample consisted of 2,826 individuals who reported being military veterans. Receipt of a physician skin examination was measured using a single question that asked participants whether they had ever had all of their skin from head to toe checked for cancer by a dermatologist or some other kind of doctor.

**Results:**

Less than a third (30.88%) of participants reported ever having a physician skin examination. Factors positively associated with receipt of a physician skin examination in a multivariable logistic regression analysis included: older age, greater educational level, non-Hispanic white race/ethnicity, having TRICARE (military) health insurance, greater skin sensitivity to the sun, and engagement in more sun protection behaviors.

**Conclusions:**

The majority of military veterans have never been screened for skin cancer by a physician. Screening rates were higher among individuals with one or more skin cancer risk factors. Future research is warranted to test targeted skin cancer screening interventions for this at risk and understudied population.

## Introduction

Military veterans are an important yet understudied group at increased risk for skin cancer, including fatal melanomas [1]. This elevated risk is likely attributable to numerous factors, including: intermittent high ultraviolet radiation exposure from the sun during active duty; barriers to engaging in sun protection during military operations; high sunburn rates; elevated exposure to in-flight cosmic ionizing radiation; and over-representation of older White men among veterans [1]. Thus, there have been recommendations for military veterans to receive periodic, routine physician screenings for skin cancer [1–3]. The U.S. Preventive Services Task Force has not recommended skin cancer screening for the average-risk U.S. population, despite some controversy and experiences of other countries that have administered national skin cancer screening programs such as the German SCREEN program [4–7]. Little is known about receipt of skin cancer screening by military veterans. The primary goal of the current study was to examine the prevalence and correlates of physician skin cancer screening among military veterans.

## Materials and methods

### Procedures

The data were drawn from the 2015 National Health Interview Survey (NHIS), a probability-based in-person survey of civilian, non-institutionalized U.S. adults. Detailed information regarding study methods and survey measures is available elsewhere [8]. In brief, a sample of 33,672 adults aged 18 years and older was recruited (response rate = 55.2%). The cancer-related module is only included in the NHIS every five years. The current study focused on the sample of 2,826 military veterans (who had ever served on active duty in the U.S. Armed Forces, military reserves, or National Guard) who reported no personal history of skin cancer and responded to the physician skin cancer screening items. This study was deemed exempt by the Rutgers Health Sciences Institutional Review Board.

### Measures

Veterans were asked whether they had ever served in a foreign country. Participants indicated their sex, age, highest educational attainment, race/ethnicity, region of residence in the U.S., and health insurance coverage. Drawing on prior research [9], we created a hierarchical health insurance coverage variable with the following mutually exclusive categories: TRICARE (military); private; Medicaid; other health insurance; veterans affairs (VA) health care only; uninsured (including Indian Health Service). Additional items asked individuals about their source of routine preventive health services, their level of worry about paying medical bills, their family history of skin cancer, their skin sensitivity to the sun, the number of sunburns they had in the past year, and whether they had ever used an indoor tanning device. We averaged responses to five questions about the frequency of engaging in sun protection behaviors (shade seeking, wearing a wide-brimmed hat, wearing a long-sleeved shirt, wearing long pants, and using sunscreen) when outside on warm sunny days (from 1 = *rarely* to 5 = *always*, with a separate category for individuals who reported that they do not go out in the sun) [10]. Responses to a series of question about the frequency and duration of engaging in moderate and vigorous intensity physical activities were used to categorize individuals as being sedentary, engaging in some activity, or meeting activity guidelines [11]. Participants indicated whether they had ever had a physician skin exam (“Have you ever had all of your skin from head to toe checked for cancer either by a dermatologist or some other kind of doctor?”), and if so, when they last had an exam.

### Statistical analyses

We used SAS 9.4 to carry out the analyses, which took into account the complex sample survey data that included sample weights and post-stratification adjustments. We conducted a multivariable logistic regression analysis to examine factors associated with ever having had a physician skin exam. The independent variables included in the multivariable logistic regression analysis are shown in Table 1. All percentages reported are weighted and all sample sizes are unweighted. A cutoff of *P* < 0.05 was used to determine statistical significance.

**Table 1:** Sample characteristics, physician skin exam screening rates, and multivariable correlates of receipt of a physician skin exam among 2,826 U.S. military veterans, 2015 National Health Interview Survey.

Variable	Sample %	Ever Had Physician Skin Exam %	AOR (95% CI)^a^
Served in foreign country			
No	46.38	29.99	1 [Reference]
Yes	53.62	31.77	1.06 (0.84–1.33)
Sex			
Male	91.31	30.90	1 [Reference]
Female	8.69	30.62	1.30 (0.84–1.99)
Age in years			
18–39	12.07	13.72	1 [Reference]
40–49	11.95	20.69	**1.64 (1.03–2.59)**
50–64	24.34	27.50	**2.29 (1.49–3.54)**
≥65	51.63	38.84	**4.18 (2.63–6.62)**
Education level			
≤ High school	31.68	24.46	1 [Reference]
Some college	38.34	27.40	1.29 (0.99–1.68)
≥ College graduate	29.98	42.16	**1.94 (1.43–2.63)**
Race/ethnicity			
Non-Hispanic white	79.63	35.37	1 [Reference]
Non-Hispanic black	13.10	12.70	**0.33 (0.22–0.50)**
Other	7.27	14.41	**0.37 (0.22–0.61)**
Geographic region			
Northeast	14.07	33.97	1 [Reference]
Midwest	23.27	24.85	**0.66 (0.45–0.98)**
South	40.30	32.22	1.13 (0.77–1.64)
West	22.36	32.79	0.96 (0.66–1.39)
Health insurance			
TRICARE	12.88	40.24	1 [Reference]
Private	49.63	31.01	**0.62 (0.43–0.90)**
Medicaid	6.07	26.12	0.79 (0.44–1.41)
Other	21.59	33.03	**0.57 (0.37–0.86)**
VA health care only	6.31	20.63	0.61 (0.35–1.06)
Uninsured	3.52	9.52	**0.37 (0.15–0.87)**
Source of preventive health care			
Doctor office or HMO	62.04	34.23	1 [Reference]
Clinic or health center	24.56	27.66	0.87 (0.65–1.15)
Hospital or other place	8.03	27.47	1.19 (0.76–1.85)
Nowhere	5.36	11.29	**0.36 (0.18–0.73)**
Worry about medical bills			
Not at all worried	71.10	33.31	1 [Reference]
Somewhat worried	22.93	25.81	0.86 (0.65–1.14)
Very worried	5.97	20.58	1.02 (0.63–1.65)
Family history of skin cancer			
No	57.75	27.79	1 [Reference]
Yes	42.25	35.05	1.12 (0.88–1.43)
Skin sensitivity to the sun			
Turn darker, no sunburn	39.64	23.35	1 [Reference]
Don’t go out in the sun	7.87	29.55	1.07 (0.57–2.01)
Mild sunburn	23.97	33.71	1.26 (0.94–1.68)
Moderate/severe sunburn	28.52	39.41	**1.46 (1.08–1.97)**
No. of past year sunburns			
0	75.59	30.29	1 [Reference]
1	12.46	34.37	1.36 (0.93–1.74)
≥2	11.95	30.63	1.06 (0.75–1.48)
Ever indoor tanned			
No	90.03	30.91	1 [Reference]
Yes	9.97	30.03	1.08 (0.74–1.58)
Sun protection composite score			
1.0–2.0	30.43	24.56	1 [Reference]
2.1–3.0	41.01	30.22	1.09 (0.82–1.44)
3.1–4.0	16.92	41.05	**1.64 (1.17–2.30)**
4.1–5.0	3.18	49.80	**2.37 (1.29–4.35)**
Don’t go out in the sun	8.46	28.73	1.07 (0.56–2.05)
Physical activity level			
Sedentary	35.32	26.10	1 [Reference]
Some activity	19.99	31.79	1.26 (0.93–1.70)
Meet/exceed activity guidelines	44.69	34.31	**1.43 (1.08–1.89)**

Data Source: National Center for Health Statistics, National Health Interview Survey, 2015 Abbreviations: VA, veterans affairs; HMO, health maintenance organization; AOR, adjusted odds ratio; CO, confidence interval.

^a^ Results of a multivariable logistic regression analysis examining correlates of ever having a physician skin exam. Statistically significant adjusted odds ratios are denoted with bold font. Cases with missing data (2.5% or fewer for each covariate) were excluded from analyses.

## Results

Consistent with the U.S. veteran population, the sample was comprised largely of non-Hispanic white, middle-aged and older men (See Table 1). Almost two-thirds of veterans had either TRICARE or private health insurance. Fewer than a third (30.88%) of veterans reported ever having a physician skin exam. Among those reporting having a physician skin exam, half (51.60%) indicated that they had this exam in the past year. Results of the multivariable logistic regression analysis identified numerous factors that were significantly associated with a higher rate of physician skin cancer screening, including: older age; a higher level of education; being non-Hispanic white; living in the Northeast compared to the Midwest; having TRICARE health insurance; receiving preventive health care at a doctor’s office or health maintenance organization; having skin that is more sensitive to the sun; engaging in more sun protection behaviors; and being more physically active.

## Discussion

Just under a third of military veterans in the current study reported that a physician had ever screened them for skin cancer. This figure is higher than the overall rate of 21.3% in the U.S. population in 2015 [10]. Higher rates of screening were found among individuals with one or more skin cancer risk factors, which is consistent with research conducted with non-veteran populations [10,12].

Screening rates were higher among more physically active individuals, who are at elevated risk for melanoma, which has been attributed to increased ultraviolet radiation-related skin damage from outdoor physical activity [13]. Skin cancer screening rates varied by veterans’ insurance status, with the highest screening rates among those with TRICARE insurance, which covers skin cancer exams, and the lowest rates among uninsured veterans. This finding is consistent with prior research reporting higher rates of provision of other preventive care services in VA medical facilities compared to non-VA settings [14]. The higher physician skin cancer screening rate among individuals who reported more frequently engaging in sun protection behaviors may reflect these individuals’ greater awareness and motivation with regard to skin cancer prevention. In general, however, previous research has shown that military veterans may not perceive themselves as being at increased risk for skin cancer [15,16]. In a study of military veterans who had been deployed to Iraq or Afghanistan, 63% reported having a sunburn during deployment and 29% reported that they noticed a changing mole after deployment, both of which are skin cancer risk factors. However, only 23% reported that the U.S. military made them very aware of the risks of skin cancer [17].

The strengths of this study include the use of a probability-based sample with a sample size of nearly 3,000 military veterans. The limitations include an inability to stratify results according to military branch, lack of information regarding additional potentially relevant skin cancer risk factors (e.g., lifetime history of sunburns), and the self-report nature of the data, which is subject to recall bias. However, several reports note recall bias for melanoma-related factors such as skin examinations and sunburns to be minimal [18–21]. Overall, the study results provide valuable insights on skin cancer screening rates among at-risk military veterans and opportunities for improvements.

## Conclusions

Future research is warranted to test clinician screening interventions targeted for military veterans who are at increased risk for skin cancer. Further, such research should be accompanied by systematic efforts to improve skin cancer prevention, treatment, and outcomes among military service members and veterans, such as that supported by the Department of Defense Melanoma Research Program [22].
